# Application of health self-management intervention program for metabolic syndrome patients in the bereaved population following the Wenchuan earthquake

**DOI:** 10.3389/fpubh.2023.1277389

**Published:** 2023-12-07

**Authors:** Ma Lihua, Jiang Xiaolian, Wang Song, Jiang Ning

**Affiliations:** ^1^West China School of Nursing/West China Hospital, Sichuan University, Chengdu, China; ^2^The First Hospital of Lanzhou University, Gansu, China

**Keywords:** metabolic syndrome, bereavement, self-management, intervention, RCT - randomized controlled trial

## Abstract

**Background:**

The destructive Wenchuan earthquake has led to approximately 800,000 people being bereaved. In the previous cross-sectional study, we explored the long-term incidence of Metabolic Syndrome (MS) and studied its influencing factors among the bereaved population 12 years after the Wenchuan earthquake. Chronic disease self-management has become a recognized public health service. Studies have shown that demographic and genetic factors, stress, geographical environment, society, culture, dietary habits, lifestyle, and other aspects influence MS. Due to the Wenchuan earthquake being a serious stress event, the implementation of targeted interventions should be discussed further.

**Objectives:**

To verify the effect of applying a self-management intervention program for patients with MS among the bereaved population following the Wenchuan earthquake.

**Design:**

A randomized controlled trial (RCT) design was adopted.

**Participants:**

A total of 132 bereaved patients with MS following the Wenchuan earthquake constituted the sample.

**Methods:**

The study was based on the Cognitive–Phenomenological–Transaction, Chronic Disease Self-Management Program, and Patient Empowerment Conceptual Model, which combined with the latest evidence-based guidelines, were used to systematically evaluate cross-sectional results of this study that were used to construct a stress management-based health self-management intervention program and MS health self-management manual for bereaved patients with MS following the Wenchuan earthquake. In addition, we revised and completed a health self-management intervention program and health self-management manual for patients with MS by using the expert consultation method. General data were collected prior to intervention (T0). We collected the patients’ MS disease-related physiological indicators before intervention (T0), after intervention (T1), and 2 months after intervention (T2). EipData3.1 software was used to input data in duplex and duplicate, and SPSS22.0 software was used for statistical analysis.

**Results:**

The variance analysis showed that the total score of healthy self-management behavior and the score of diet management, exercise management, drug management, and emotional management have intergroup effects, time effects, and group–time interaction effects (*p* < 0.05). When the differences between groups were further compared, we found that the total score and the score of six dimensions (excluding disease self-monitoring management) were higher than those of the control groups at T1 and T2, and the differences were statistically significant (*p* < 0.05).

**Conclusion:**

The intervention program of healthy self-management for patients with MS who come from bereaved families following the Wenchuan earthquake can effectively improve patients’ health self-management behaviors.

## 1. Introduction

Earthquake is a natural disaster with great destructive power (1). China is a developing country with frequent earthquakes, and it is also one of the countries with the most casualties due to earthquakes (2). Wenchuan earthquake in 2008 was not only the most destructive earthquake in China after the Tangshan earthquake in 1976, but also it is one of the most destructive earthquakes in the world in the first decade of the new century (3). In the earthquake, 69,227 people were killed, 374,643 people were injured, and 17,923 people were missing, bringing direct economic losses of 845.1 billion yuan to the Chinese people. According to statistics, Wenchuan earthquake caused a total of more than 80,000 people missing or died. According to the calculation of about 10 relatives of each deceased, the earthquake caused a total of about 800,000 people bereaved (4). Bereaved people experience property loss, burial of themselves/others, injury, disability, death of relatives, interruption of medical and health services, changes in production activities and lifestyles, as well as the pain of losing relatives and the destruction of family structures and functions, and many other earthquake-related disasters. If the population is in a state of continuous stress response for a long time, it will often induce a series of neuroendocrine reactions, mainly manifested as sympathetic nerve excitation and increased secretion of hypothalamic–pituitary–adrenal cortex (5), which makes corticotropin-releasing hormone Elevated levels, the increase of these hormones is closely related to body obesity and elevated blood sugar, blood pressure, blood lipids, etc. adverse effects. At present, most of the research on the health problems of the people after the earthquake in my country is limited to physical trauma and mental and psychological aspects. More than 80% of the researches focus on 3 months to 2 years after the earthquake drastically reduced (6).

Metabolic syndrome (MS) is a group of clinical syndromes mainly manifested by metabolic disorders such as central obesity (visceral obesity), insulin resistance, glucose metabolism, lipid metabolism and hypertension (7). MS is one of the escalating public health problems in most countries and regions in the world, with its prevalence ranging from 13.1 to 43.6%, and showing an increasing trend year by year (8). Studies have found that MS is not only a risk factor for cardiovascular disease (CVD), diabetes and chronic kidney disease (9–11), but also significantly increases prostate cancer, breast cancer, leukemia, pancreatic cancer, colorectal cancer, and liver cancer. And other cancer patients, resulting in a 33% increase in overall cancer mortality (12). MS is closely related to a variety of chronic diseases, causing each other to form a vicious circle, which seriously damages people’s health, reduces the quality of life, and brings a heavy economic burden of disease to families and society.

MS is a process of accumulation of chronic damage to body functions, and is the result of decompensation of the body in response to various negative factors. It is a preventable, controllable, reversible and treatable disease. Relevant studies have found that cognitive behavioral therapy, exercise, lifestyle management, self-management education, and coping style guidance can alleviate patients’ anxiety, depression and other negative emotions, master chronic disease knowledge and improve self-efficacy, thereby improving patients’ blood sugar, blood lipid levels (13–15). A meta-analysis of 8 randomized controlled trials showed that lifestyle interventions can effectively slow the progression of MS disease, improve waist circumference (Waist Circumference, WC), blood pressure, fasting plasma glucose (Fasting Plasma Glucose, FPG) and triglycerides (Triglyceride, TG) (16).

The previous cross section explored the long-term incidence of MS and studied its influencing factors among the bereaved population 12 years after the Wenchuan earthquake. The research based on influencing factors and constructed a intervention program, which is a multi-ethnic culturally adaptable health self-management. This research provides a reliable basis and methodological reference for the systematic development of the health management of bereaved population in China, as well as established relevant guidelines and consensus especially for the MS patients in multi-ethnic gathering areas after disasters. The incidence of MS among the bereaved in the Wenchuan earthquake was 18.5% in cross section. However, there is no relevant research on the dynamic change of MS incidence in bereaved groups after disasters, so the incidence of MS in bereaved groups in Wenchuan earthquake cannot be compared with that in the same population under the same environment. Some scholars adopted the multistage and multilayer cluster sampling method in Sichuan and took CDS as the diagnostic standard. Up to October 2007, a total of 3,511 people were surveyed, and the prevalence of MS was 11.5% (male 14.3%, female 9.4%). The survey time was the closest to the prevalence of MS in Sichuan before the Wenchuan earthquake in 2008. It is suggested that the incidence rate in this study is much higher than that in the same area before the earthquake.

## 2. Methods

### 2.1. Design

A randomized controlled trial (RCT) design was adopted.

### 2.2. Research objects

#### 2.2.1. Target groups

A cross-sectional study in Yingxiu Town and Beichuan County, the severely stricken area of the Wenchuan earthquake, enrolled confirmed MS patients who were bereaved by the earthquake.

#### 2.2.2. Sampling standards

Inclusion criteria: ① MS patients diagnosed in a cross-sectional study; ② 18 years old and above; ③ permanent population (residential time more than 6 months per year); ④ ability to communicate normally and have basic literacy skills (*researchers are able to read Chinese characters and understand their meaning accurately*); and ⑤ provide informed consent and participate voluntarily.

Exclusion criteria: ① pregnant women; ② hearing impairment; ③ inability to communicate normally; ④ having cognitive impairment or mental illness.

### 2.3. Participants

In the first stage, 8 rural communities with similar economic conditions were selected as the target communities for sampling to facilitate the sampling. In order to avoid contamination, 6 communities in Yingxiu Town (*It was 11 kilometers from the epicenter*); 2 communities in Beichuan County (*It is about 132 km from the epicenter, but suffered great losses*) were the control communities.

In the second stage, paper strips with the names of the 6 village committees in Yingxiu Town were placed into an opaque container, and then they were taken twice in order. The two communities of the intervention group selected. There were 118 MS patients in the two villages.

In the third stage, the intervention group and the control group were selected from the 4 research communities selected in the first and second stages. The random sampling method was performed using Stata/MP14.0 software to generate a random number generator according to the ratio of experimental group: control group = 1:1 for randomized sample extraction.

Blind method: data collectors, data entry and statistical analysis personnel were blinded.

### 2.4. Intervention content and methods

Patients in the control group received routine chronic disease management in village clinics/township health centers. After baseline data collection, patients who were interested in participating in the study after the last data collection were informed that they could get a copy of the “Handbook” and corresponding health consultations for free. On the basis of routine management in the control group, patients in the intervention group received MS health self-management education intervention, mainly including the following:

1. Group teaching: a total of 8 times, once a week, for 8 consecutive weeks. The specific content and arrangement are shown in Table 1. The patients and family members were required to participate together to preview the relevant parts of the self-study manual before listening to the class. Teaching method: group multimedia lectures, 10–15 people in each group, 3 ~ 5 people discussed and shared after the lecture, and individual tutoring was conducted and questions answered according to the needs of the intervention subjects.
2. Log records: MS health self-management logbooks were distributed to guide patients to record daily so that patients could reflect on self-management, and it was also convenient for researchers to find problems and provide timely feedback.
3. WeChat online help/telephone follow-up: WeChat online help was used to solve the problems of the intervening subjects online at any time during the course and within 4 weeks after the end of the course. At the same time, the intervention subjects received 4 weeks of telephone/face-to-face follow-up once a week at the end of the course. The telephone/face-to-face follow-up was carried out according to the follow-up record sheet. The content mainly included assessing the patient’s self-management practice at various stages, checking the completion of expected management goals, giving patients self-management support and encouragement, and helping patients solve the practice process difficulties and problems encountered.

**Table 1:** Health self-management intervention program for patients with metabolic syndrome.

Subjects	Purpose	Contents	Methods
1. Project start 2. Transfer of MS patients diagnosed for the first time	1. Understand the purpose and significance of the project. 2. Establish a partnership with the target of intervention. 3. Help patients with MS diagnosed for the first time transfer to doctors in village clinics or township health centers.	1. Distribute the “Manual on the Health Self-management of MS Patients in the Wenchuan Earthquake Bereavement” (hereinafter referred to as the “Manual”). 2. Have intervention objects introduce themselves and become familiar with each other. 3. Introduce project. 4. Provide feedback of the baseline assessment results of various metabolic indices of MS patients. 5. Help patients with MS diagnosed for the first time transfer to doctors in village clinics or township health centers. 6. Establish a WeChat group to answer questions online and provide instant help.	1. The researcher introduces himself. 2. Intervention objects introduce themselves and meet each other. 3. Project introduction, emphasizing the significance and importance of the project. 4. Distribute the “Manual” to explain the usage and requirements for pre-class preparation and after-class review. 5. Feedback the baseline assessment results of various metabolic indices of MS patients. 6. Help patients with MS diagnosed for the first time transfer to doctors in village clinics or township health centers. 7. Establish a WeChat group, inform the purpose of the group, and let participants know that if they have any questions, they can ask online at any time.
Lesson 1 Overview of MS disease-related knowledge and health self-management	1. Master MS disease related knowledge, MS risk factors and prevention. 2. Recognize the importance of MS health self-management, six management strategies (stress/emotion, diet, daily life, exercise/weight, drug management and self-monitoring). 3. Develop self-management goals for MS health.	1. Feedback on qualitative interviews and baseline assessment of patients’ knowledge of MS prevention and treatment. 2. Study the first part to the third part of the “Handbook.” 3. Explain the importance of MS health self-management. 4. Formulate health self-management goals. 5. Evaluation and feedback on the effect of this lecture by the intervention objects.	1. Feedback on the qualitative interview of MS prevention knowledge mastery and baseline “MS Prevention Knowledge Scale” evaluation results. 2. PPT explains the first to third parts of the “Handbook” (the definition of metabolic syndrome, diagnostic criteria and hazards; MS risk factors; MS prevention and treatment). 3. Explain the importance of health self-management for MS patients. 4. Jointly develop health self-management goals with the intervention targets. 5. Evaluation and feedback on the effect of this lecture by the intervention objects.
Lesson 2 Stress/emotion management 1 (Daily life)	1. Understand the source, classification and stress response of daily life stress. 2. Recognize the importance of stress management. 3. Master the daily stress management strategy. 4. Master common pressure relaxation techniques.	1. Review the essentials of MS disease knowledge. 2. Feedback cross-sectional research on the psychological influencing factors of MS, the influencing factors of emotional management in qualitative interviews and the misunderstandings, and the baseline assessment results of emotional management in “Health Self-Management Behavior of MS Patients.” 3. Study the content of daily life stress management in Part 4, Section 5, “Stress/Emotion Management” of the “Handbook.” 4. Demonstrate common training techniques for stress relaxation. 5. Evaluation and feedback on the effect of this lecture by the intervention objects.	1. Review the essentials of MS disease knowledge. 2. Provide feedback on cross-sectional research on the psychological influencing factors of MS disease, qualitative interview emotional management influencing factors and misunderstandings; and feedback on the baseline evaluation results of emotional management in “Healthy Self-Management Behavior of MS Patients.” 3. PPT explains the “Manual” Part 4, Section 5 “Stress/Emotion Management” Daily Life Stress/Emotion Management: ① Correctly understand stress and stress response: the source of daily stress (life, environment, society, etc.) and stress response (physiology, psychology, behavior); acceptance of stress/emotion exists objectively ② Correctly manage stress and stress response: the importance of stress/emotion management to health; adjustment of bad cognition; establish a positive and healthy outlook on life and values; vent bad emotions; actively cope with stress ③ Actively seek social support and use social resources: seek relatives, friends, psychologists, etc. to express their pressure/emotions; use social resources to express emotions and obtain information, financial support, etc. ④ Common relaxation skills training: breathing training, meditation exercises and progressive muscle relaxation training 4. Audio/video demonstration of stress relaxation training (breathing training, meditation exercises and progressive muscle relaxation training) methods. 5. Evaluation and feedback on the effect of this lecture by the intervention objects.
Lesson 3 Stress/emotion management 2 (Bereavement crowd)	1. Recognize the impact of earthquake stress on the health of bereaved families. 2. Understand grief and grief reactions. 3. Master stress/emotion management strategies (grief coping, family management and posttraumatic growth strategies). 4. Develop stress self-management plans and goals.	1. Review the essentials of daily stress/emotion management. 2. Study the content of stress/emotion management for bereavement groups in Part 4, Section 5, “Stress/Emotion Management” of the “Handbook.” 3. Develop stress management plans and goals. 4. Evaluation and feedback on the effect of this lecture by the intervention objects.	1. Review the essentials of daily stress/emotion management. 2. PPT explains the “Manual” Part IV, Section 5 “Stress/Emotion Management” Pressure/Emotion Management of Bereavement Groups: ① Relevant knowledge: bereavement grief and grief reaction; the impact of earthquake stress on the health of bereaved people. ② Stress/emotion management strategy □Sorrow coping strategies: accept facts, think positively (combining Qiang and Tibetan religious beliefs); seek emotional companionship, etc. □Family business management: reorganization/remarriage family business strategy; family relationship improvement strategy; effective family support and utilization strategy; list of tasks for intervention targets □Growth management: Cognitive reconstruction, optimistic face, accomplish things that can bring a sense of accomplishment to oneself, learn to appreciate life, increase interpersonal communication, increase new possibilities (interests, job opportunities, etc. combined with local development) and enhance personal strength (strong, independent, brave) 3. Group discussion: stress/emotion management challenges and problems. 4. Experience sharing: stress/emotion management experience. 5. Develop stress/emotion management plans and goals with the intervention target. 6. Intervention objects evaluate and feedback on the effect of this lecture.
Lesson 4 diet manage	1. Master the purpose, methods and content of diet management. 2. Develop a diet management plan.	1. Review the essentials of stress/emotion management. 2. Feedback qualitative research on some of the influencing factors and misunderstandings of diet self-management, the baseline assessment results of dietary management in “Health Self-Management Behavior of MS Patients.” 3. Study “Diet Management” in Part 4, Section 1 of “Handbook.” 4. Formulate diet management plans and goals. 5. Evaluation and feedback on the effect of this lecture by the intervention objects.	1. Review the essentials of stress/emotion management. 2. Feedback on the qualitative interviews on some of the influencing factors and misunderstandings of diet management, and the baseline assessment results of diet management in “Health Self-Management Behavior of MS Patients.” 3. PPT explains the contents of Section 1 of Part Four of the “Handbook” (Diet Management Principles, Three Major Nutrients, Diabetes and Hypertension Dietary Points, Tips for Dietary Cooking). 4. Group discussion: problems and solutions in self-management of diet. 5. Experience sharing: experience in self-management of diet. 6. Develop diet self-management plans and goals with the intervention targets. 7. Evaluation and feedback on the effect of this lecture by the intervention objects.
Lesson 5 1. Daily life management 2. Exercise/weight management	1. Master the purpose, methods, content and precautions of daily life, exercise/weight management. 2. Develop daily life and exercise management plans.	1. Review the main points of diet management. 2. Provide feedback on cross-sectional research on the impact of daily life (smoking, drinking, sleep quality) and physical exercise on MS, qualitative research on the weak links and misunderstandings of daily life management; the baseline of daily life and exercise management in “Health Self-Management Behavior of MS Patients” evaluation result. 3. Study “Manual” Part 4, Section 2 “Daily Life Management” and Section 3 “Exercise/Weight Management.” 4. Develop self-management plans and goals for smoking, drinking, sleep and exercise. 5. Evaluation and feedback on the effect of this lecture by the intervention objects.	1. Review the main points of diet management. 2. Provide feedback on cross-sectional research on the impact of daily life (smoking, drinking, sleep quality) and physical exercise on MS, share the results of qualitative research results in patients with misunderstandings about drinking and physical exercise. 3. PPT explains the contents of Section 2 and Section 3 of the fourth part of the “Handbook”: daily life management and sports management: □ The hazards of smoking (including snuff), the relationship between smoking and metabolic syndrome, the benefits of quitting, tips for quitting, how to deal with difficulties in quitting □ The harm of drinking, the relationship between drinking and metabolic syndrome, the misunderstanding of drinking to lower blood sugar, the contraindications of drinking and drugs, and the coping strategies of drinking; tips for improving sleep quality □ Importance and significance of exercise/weight management, calculation of normal weight range, exercise intensity evaluation method, exercise method, exercise precautions, prevention and coping strategies of exercise hypoglycaemia, exercise misunderstanding 4. Group discussion: problems and solutions in daily life management and sports management. 5. Experience sharing: daily life and exercise self-management experience. 6. Work with the intervention subjects to develop self-management plans and goals for sports and daily life. 7. Intervention objects evaluate and feedback on the effect of this lecture.
Lesson 6 Drug management	1. Recognize the importance of drug management for MS control. 2. Master the names, usage, effects and precautions of commonly used antihypertensive drugs, lipid-lowering drugs, and hypoglycaemic drugs. 3. Master the method of insulin injection. 4. Develop a drug management plan.	1. Review the essentials of daily life management and exercise management. 2. Provide feedback on the evaluation results of baseline drug management of intervention subjects, weaknesses and misunderstandings of qualitative research drug management. 3. Study “Drug Management” in Part IV, Section 4 of the “Handbook.” 4. Formulate drug management plans and goals. 5. Evaluation and feedback on the effect of this lecture by the intervention objects.	1. Review the essentials of daily life management and exercise/weight management of MS patients. 2. Provide feedback on the results of baseline drug management and qualitative research on weak links and misunderstandings in drug management. 3. PPT explains the “Drug Management” in Part IV, Section 4 of the “Handbook”: □ Importance of drug management □ The names, usage, effects and precautions of commonly used antihypertensive drugs, lipid-lowering drugs, and hypoglycaemic drugs □ Misunderstanding of drug management □ Medication management strategy □ Demonstrate insulin injection method 4. Group discussion: problems and solutions in drug management. 5. Experience sharing: drug management experience. 6. Jointly formulate drug management plans and goals with the intervention targets. 7. Evaluation and feedback on the effect of this lecture by the intervention objects.
Lesson 7 Self-monitoring (1) (theory)	1. Recognize the importance of metabolic indicators and monitoring of complications. 2. Understand the time, significance and control objectives of blood glucose, blood pressure, weight and waist measurement.	1. Review the main points of medication management. 2. Provide feedback on the self-monitoring baseline assessment results and qualitative research on the weaknesses and misunderstandings of self-monitoring. 3. Study the self-monitoring in Section 6 of Part IV of the “Handbook.”	1. Review the main points of medication management. 2. Provide feedback on self-monitoring baseline assessment results, qualitative research on the weaknesses and misunderstandings of self-monitoring. 3. PPT explains the content of “Self-Monitoring” in Part 4, Section 6 of the “Handbook”: ◇ □ Importance of monitoring of MS metabolic indicators ◇ □ MS control target value of each metabolic index ◇ □ Monitoring time point and significance of blood sugar, blood pressure, etc. ◇ □ The difference between blood glucose monitoring and HbA1c monitoring ◇ □ Blood glucose, blood pressure, waist circumference and weight monitoring frequency and precautions Importance, content and specific monitoring items of MS complications monitoring
Lesson 8 Self-monitoring (2) (Measurement methods)	1. Master the measurement methods of each component of MS. 2. Master the use of health management log records.	1. Review the relevant knowledge of MS self-monitoring. 2. Demonstrate the measurement method of each component of MS. 3. Explain how to use the health self-management log record sheet. 4. Develop self-monitoring plans and goals. 5. Evaluation and feedback on the effect of this lecture by the intervention objects.	1. Review the essentials of MS self-monitoring knowledge. 2. Demonstrate how to measure blood sugar, blood pressure, weight and waist circumference. 3. Explain how to use the health self-management log record sheet. 4. Group discussion: self-monitoring problems and solutions. 5. Experience sharing: self-monitoring experience. 6. Jointly develop self-monitoring plans and goals with the intervention targets. 7. Evaluation and feedback on the effect of this lecture by the intervention objects.
Intervention follow-up 1 month	1. Check the management plan and the completion of management objectives. 2. Support and encourage intervention targets to implement management strategies. 3. Help the intervening object to solve the difficulties and problems in the implementation of self-management.	1. Check the implementation of the self-management plan and the achievement of management objectives. 2. Help the intervening object to solve the difficulties and problems encountered in the implementation of self-management. 3. Encourage and support patients to implement management strategies.	According to the follow-up record sheet, follow up once a week by phone or face-to-face; combined with online communication and help of WeChat group.

### 2.5. Intervention effect evaluation indicators and evaluation tools

#### 2.5.1. Evaluation time

In this study, the intervention evaluation time was set at baseline (T_0_), at the end of the intervention (8 weeks of teaching plus 4 weeks of follow-up, T_1_), and 2 months after the end of the intervention (T_2_) to evaluate the effect of the intervention and to verify the metabolic syndrome at different follow-up times.

#### 2.5.2. Evaluation tools and methods

1. General information survey form.

① Demographic data: gender, age, marital status, education level, religious beliefs, family monthly income *per capita*, medical payment method, occupation, etc.

② Earthquake traumatic experience: self-buried/injured/disability in the earthquake, family property loss, witnessing the burial/injury/death of others in earthquake, loss of specific relatives, number of bereavements, and remarriage/rebirth.

③ Disease-related information: family genetic history, etc.

1. Self-management Behavior Scale for Patients with MS.

The scale is based on the chronic disease self-management theory (17). Identification of metabolic syndrome using phenotypes consisting of triglyceride levels with anthropometric indices in Korean adults [J]. In 2019, Dr. Ni Zhihong from West China Hospital of Sichuan University developed a “Metabolic Synthesis” based on the compilation of the chronic disease education and research center of Stanford University and other scholars (18). Self-management Behavior Scale, which can comprehensively evaluate the MS self-management behavior of community residents, including 7 dimensions (diet management, exercise/weight management, daily life style management, medication management, emotional management, self-monitoring), 36 items, using Likert 5-level scoring method, the option scores are 1 = never, 2 = occasionally, 3 = sometimes, 4 = frequently, 5 = always, the total score is 180 points, the more the score is High indicates that the patient’s self-management behavior is better. The Cronbach’s α coefficient of the scale is 0.868, and the test–retest reliability is 0.957.

#### 2.5.3. Pre-test

To determine the performance of the MS patients’ health self-management research tool and the feasibility of the program, the researchers used Stata. The MP14.0 software random number generator randomly selected 30 MS patients and randomly assigned them to the control group (*n* = 15) and the self-management intervention group (*n* = 15). The control group received chronic disease management in conventional village clinics/township hospitals, and the self-management education group received MS health self-management interventions and conventional village clinic chronic disease management. Patient-related data were collected before and after the intervention. The results show that the overall feasibility of the research program is good, the intervention process (8 weeks of teaching plus 4 weeks of follow-up) and the process of data collection are culturally adaptable and easy to communicate, and the questionnaire expression is clear and easy to understand. In addition, after collecting baseline data, 15 patients in the control group were re-evaluated by the MS Patient Self-Management Behavior Scale at 2 week intervals. The test–retest reliability, Cronbach’s coefficient and test–retest reliability of the scale met the statistical requirements.

#### 2.5.4. Data collection

1. Data collectors: 2 local medical undergraduates in fourth grade were recruited and unified training was conducted (1 student was responsible for patient physiological index measurement, and the other student was responsible for filling the patient questionnaire). Measurement methods and precautions were explained to ensure the accuracy of data collection.
2. Data collection location: ① questionnaire data were collected at home; ② basic disease data (anthropometric and physiological data) were measured on an empty stomach in each village clinic/township health center.
3. How to fill in the questionnaire: the respondents should have filled in the questionnaire by themselves. To ensure the accuracy and completeness of the information, when the questionnaire was collected, it was checked on the spot for any missing or questionable items. The respondents were asked to fill in and verify the questionnaire and then take it back after verification.
4. Collection method of disease-related physiological indicators: same as the current situation investigation part.
5. Data collection time: a formal intervention trial was conducted from March to July 2021, and relevant data of patients at baseline (T_0_), end of intervention (T_1_) and 2 months after the end of intervention (T_2_) were collected.

#### 2.5.5. Statistical analysis

Statistical description: Counting data are described by frequency and composition ratio; normal distribution of measurement data is described by mean ± standard deviation, and skew distribution is described by median and interquartile range.

Statistical inference: SPSS 22.0 software was used to perform statistical analysis on the data. According to the level of α = 0.05, the *p* value is a two-sided probability for statistical inference. ① Comparison of enumeration data between groups: chi-square test or Fisher’s exact probability method was used. ② Comparison of measurement data between groups: comparison of means of normal distribution: if the variances were uniform, the group *t* test was performed, and if the variances were not uniform, the *t*’ test or Wilcoxon rank sum test was performed. Wilcoxon rank sum test was performed for comparison of nonnormal distribution means. ③ Intragroup time effect and between-group effect comparison of each index: repeated measurement data analysis of variance or generalized mixed benefit model analysis was performed.

### 2.6. Ethical issues

This study strictly follows the biomedical ethics code (No: 965). The research plan was sent to the Ethics Committee of West China Hospital of Sichuan University for approval.

## 3. Results

### 3.1. Baseline situation of study subjects

#### 3.1.1. Information collection

A total of 330 MS patients who were bereaved in the Wenchuan earthquake were established based on the diagnostic criteria of the “Guidelines for the Prevention and Treatment of Type 2 Diabetes in China (2017)” (19). All study subjects completed the T_0_ baseline data collection; T_1_ collected a total of 127 valid data points, with a loss to follow-up rate of 3.78%; T_2_ collected a total of 124 valid data points, with a loss to follow-up rate of 2.27%; the total loss to follow-up rate was 6.06%.

#### 3.1.2. General information of research objects

After normality testing, the ages of the control group and the intervention group did not follow a normal distribution (Kolmogorov–Smirnov test, *p* < 0.05), and the 25th percentile, median, and 75th percentile were used to describe the two age distribution characteristics of the groups. In addition, the demographic data and the remaining variables of the earthquake trauma experience between the two groups were count data, which were described by frequency and composition ratio. According to the data characteristics, the statistical method used Pearson’s chi-square test or Fisher’s exact test. There was no significant difference between the basic demographic data and earthquake trauma experience data of the two groups of patients (*p* > 0.05) see Table 2.

**Table 2:** Baseline characteristics of general data of research subjects (*n* = 132).

Index	Number (%)		*χ*^2^/U	*p*
	Intervention group (*n* = 66)	Control group (*n* = 66)		
Age (years old)	51.2	52.5	Z = 1.100	0.271^#^
Median (*P25,P75*)	(40.8, 64.2)	(45.8,60.2)		
Gender				
Male	30 (45.5)	28 (42.4)	1.939	0.191*
Female	36 (54.5)	38 (57.6)		
Education				
Elementary school and below	42 (63.6)	47 (70.3)	1.011	0.799*
junior high school	18 (27.3)	14 (21.1)		
High school / technical secondary school and above	6 (9.1)	5 (7.7)		
Marital status				
Unmarried	3 (4.5)	1 (1.5)	5.125	0.401^**^
Married	53 (80.4)	53 (80.4)		
Divorced	2 (3.0)	1 (1.5)		
Widowed	8 (12.1)	11 (16.6)		
Occupation				
Non-farmers	17 (25.8)	10 (15.2)	2.281	0.195*
Farmer	49 (74.2)	56 (84.8)		
Ethnic				
Han	40 (60.6)	48 (72.7)	2.505	0.286^**^
Qiang	22 (33.3)	14 (21.2)		
Tibetan	4 (6.1)	4 (6.1)		
Residence status				
Live alone	6 (9.1)	3 (4.5)	1.073	0.492^*^
Not living alone	60 (90.9)	63 (95.5)		
Medical expenses types				
Rural residents’ medical insurance	27 (40.9)	18 (27.3)	2.731	0.098^*^
Rural cooperative medical system	39 (59.1)	48 (72.7)		
Household monthly income *per capita*				
No fixed income	4 (6.1)	13 (19.7)	6.277	0.099^**^
≤ 1,000	6 (9.1)	5 (7.6)		
1,001 ~ 3,000	44 (66.7)	41 (62.1)		
3,001 ~ 5,000	12 (18.1)	7 (10.6)		

Remark:*, Pearson Chi-square test; **, Fisher’s Exact test.

The average age of the 132 survey subjects included in this section was 51.85 ± 12.19 years old; there were more women than men, accounting for 56.1%. The education level was mainly elementary school and below, accounting for 67.4%. The proportion of married people (including remarried) was 63.6% (12.9%). The occupation was mainly agriculture, accounting for 79.5%. The ethnic group was mainly Han nationality, accounting for 66.7%. The living status was mainly not living alone, accounting for 93.2%. The medical payment was mainly based on the new rural cooperative medical system, with a proportion of 65.9%. The family monthly income *per capita* was mainly 1,000–3,000 yuan/month/person, accounting for 64.4%. The specific demographic data of the intervention group and the control group are shown in Table 2.

In the earthquake trauma experience of the study subjects, 34.1% were buried themselves during the earthquake, 35.6% were injured themselves, 3.8% were disabled, 38.6% experienced severe or above property losses caused by the earthquake, and 85.3% were injured during the earthquake. The survey respondents witnessed others being buried, 84.1% witnessed others being injured, and 71.2% witnessed the death of others. The specific trauma experience data of the intervention group and the control group are shown in Table 3.

**Table 3:** Study subject’s earthquake trauma experience (*n* = 132).

Index	Category	Number (%)		*χ* ^2^	*p*
		Intervention group (*n* = 66)	Control group (*n* = 66)		
I was buried in the earthquake	Yes	26 (39.4)	19 (28.8)	1.652	0.199^*^
	No	40 (60.6)	47 (71.2)		
I was injured in the earthquake	Yes	28 (42.4)	19 (28.8)	2.676	0.102^*^
	No	38 (57.6)	47 (71.2)		
I was disabled during the earthquake	Yes	3 (4.5)	2 (3.0)	0.208	0.648^*^
	No	63 (95.5)	64 (97.0)		
Family members died or whereabouts are unknown in the earthquake	1 person	24 (36.4)	30 (45.5)	2.090	0.352^**^
	2 person	29 (43.9)	21 (31.8)		
	3 people and above	13 (19.7)	15 (22.7)		
Property loss	none	2 (3.0)	4 (6.1)	6.999	0.136^**^
	Mild	5 (7.6)	12 (18.2)		
	Moderate	28 (42.4)	30 (45.5)		
	severe	20 (30.3)	10 (15.1)		
	Extremely severe	11 (16.7)	10 (15.1)		
Witnessed others being buried by the earthquake	Yes	35 (53.0)	42 (63.6)	1.527	0.217^*^
	No	31 (47.0)	24 (36.4)		
Witnessing others injured by the earthquake	Yes	52 (78.8)	59 (89.4)	2.775	0.096^*^
	No	14 (21.2)	7 (10.6)		
Witnessed the death of others by the earthquake	Yes	49 (74.2)	45 (68.2)	0.591	0.442^*^
	No	17 (25.8)	21 (31.8)		

Remark:*, Pearson Chi-square test; **, Fisher’s Exact test.

### 3.2. The impact of MS self-management on patients’ health self-management behavior and disease-related metabolic indicators

The total scores of health self-management behaviors and disease-related metabolic indicators at T_0_, T_1_, and T_2_ of the two groups of patients are shown in Tables 4 and 5. The total scores of patients’ health self-management behaviors at three time points and disease-related metabolic indicators were analyzed for variance analysis and sphere test (Mauchly method) of repeated measurement data. The results of the sphere test showed *p* > 0.05, indicating that the data met the conditions of the sphere test. One-way analysis of variance was performed.

**Table 4:** Comparison of the scores of healthy self-management behaviors between the two groups (^−^*χ* ± s, *n* = 132).

Index	groups	T_0_	T_1_	T_2_	F_inter-group_ (*p*)	F_time_ (*p*)	F_Interactive_ (*p*)
Total score	Control	85.05 ± 4.06	85.61 ± 3.91	86.23 ± 3.74			
	intervention	84.14 ± 5.07	90.20 ± 4.99	93.74 ± 4.64			
	*t*	−1.136	5.874	6.605	23.912	121.617	80.945
	*p*	0.258	<0.001	<0.001	(<0.001)	(<0.001)	(<0.001)
Diet managem-ent	Control	31.74 ± 3.06	32.39 ± 2.91	32.35 ± 2.89			
	intervention	31.27 ± 3.02	33.83 ± 3.07	34.52 ± 2.95			
	*t*	−0.887	2.757	4.261	42.979	4.418	20.912
	*p*	0.376	0.007	<0.001	(<0.001)	(0.039)	(<0.001)
Exercise managem-ent	Control	7.30 ± 1.43	8.14 ± 1.10	8.52 ± 1.14			
	intervention	7.39 ± 1.33	7.61 ± 1.18	7.73 ± 1.00			
	*t*	−0.377	2.652	4.219	5.213	2.652	4.219
	*p*	0.707	0.009	<0.001	(0.026)	(0.009)	(<0.001)
Emotional managem-ent	Control	13.24 ± 1.71	13.14 ± 1.41	13.12 ± 1.53			
	intervention	13.02 ± 1.84	13.74 ± 1.94	14.26 ± 1.26			
	*t*	−0.734	2.052	4.637	4.155	15.109	27.944
	*P*	0.464	0.042	<0.001	(0.046)	(<0.001)	(<0.001)
Drug managem-ent	Control	8.00 ± 1.41	8.08 ± 1.31	7.64 ± 1.43			
	intervention	7.92 ± 1.37	8.67 ± 1.50	9.09 ± 1.46			
	*t*	−0.312	2.404	5.769	9.319	7.047	23.193
	*p*	0.755	0.018	<0.001	(0.003)	(0.002)	(<0.001)
Disease self-monit-oring	Control	9.67 ± 1.63	9.68 ± 1.64	9.85 ± 1.38			
	intervention	9.65 ± 1.65	9.88 ± 1.61	10.39 ± 1.35			
	*t*	−0.053	0.480	2.286	0.669	15.738	5.535
	*p*	0.958	0.632	0.024	(0.416)	(<0.001)	(0.006)
Other lifestyle	Control	8.91 ± 1.37	8.83 ± 1.26	9.20 ± 1.14			
	intervention	8.86 ± 1.58	9.71 ± 1.44	10.09 ± 1.59			
	*t*	−0.176	3.726	3.704	7.259	25.630	15.554
	*p*	0.861	<0.001	<0.001	(0.009)	(<0.001)	(<0.001)
Communication with physicians	Control	6.09 ± 1.21	5.88 ± 1.17	6.35 ± 0.86			
	intervention	6.11 ± 1.12	6.29 ± 1.17	6.88 ± 1.10			
	*t*	0.074	2.005	3.197	3.481	23.762	6.885
	*p*	0.941	0.047	0.002	(0.067)	(<0.001)	(0.002)

Remarks: interaction, intervention × time.

**Table 5:** Comparison of metabolic indicators between the two groups (^−^*χ* ± s, *n* = 132).

Index	groups	T_0_	T_1_	T_2_	F_inter-group_ (*p*)	F_time_ (*p*)	F_Interactive_ (*p*)
Weight	Control	73.06 ± 10.39	73.42 ± 10.11	73.74 ± 10.05			
	intervention	71.02 ± 9.45	69.50 ± 9.53	66.76 ± 7.96			
	*t*	−1.182	−2.294	−4.425	11.433	35.395	79.499
	*p*	0.239	0.023	<0.001	(0.001)	(<0.001)	(<0.001)
WC	Control	92.48 ± 8.64	92.24 ± 8.37	92.52 ± 8.05			
	intervention	91.67 ± 6.59	89.58 ± 5.76	88.62 ± 5.51			
	*t*	−0.611	−2.131	−3.240	4.096	28.988	33.742
	*p*	0.542	0.035	0.002	(0.047)	(<0.001)	(<0.001)
BMI	Control	25.91 ± 2.68	26.11 ± 2.62	26.18 ± 2.68			
	intervention	26.39 ± 2.21	25.80 ± 2.17	24.79 ± 2.06			
	*t*	1.130	−0.722	3.341	1.041	35.505	71.261
	*p*	0.260	0.472	<0.001	(0.311)	(<0.001)	(<0.001)
SBP	Control	138.03 ± 17.45	136.26 ± 17.76	137.47 ± 17.03			
	intervention	141.85 ± 20.88	138.76 ± 20.44	137.05 ± 17.35			
	*t*	1.140	0.750	−0.142	0.403	9.926	4.841
	*p*	0.257	0.455	0.888	(0.528)	(<0.001)	(0.009)
DBP	Control	83.52 ± 10.00	83.30 ± 9.65	84.48 ± 8.76			
	intervention	84.95 ± 12.05	84.48 ± 10.65	83.94 ± 9.94			
	*t*	0.746	0.668	−0.334	0.185	1.340	8.408
	*p*	0.457	0.505	0.739	(0.668)	(0.269)	(0.001)
FPG	Control	6.24 ± 1.78	6.27 ± 1.43	6.31 ± 1.36			
	intervention	6.12 ± 1.46	5.94 ± 1.17	5.83 ± 0.87			
	*t*	−0.427	−1.428	2.400	1.557	1.125	4.106
	*p*	0.670	0.156	0.018	(0.217)	(0.328)	(0.019)
TC	Control	4.30 ± 0.79	4.33 ± 0.79	4.38 ± 0.75			
	intervention	4.49 ± 0.93	4.38 ± 0.89	4.25 ± 0.81			
	*t*	1.223	0.341	−0.958	0.067	1.823	8.086
	*p*	0.223	0.733	0.340	(0.797)	(0.170)	(0.001)
TG	Control	2.26 ± 1.17	2.25 ± 1.08	2.30 ± 0.97			
	intervention	2.21 ± 0.95	2.14 ± 0.91	2.08 ± 0.86			
	*t*	−0.261	−0.608	−1.361	0.456	1.614	3.571
	*p*	0.795	0.544	0.176	(0.502)	(0.203)	(0.031)
LDL-C	Control	2.92 ± 0.75	2.92 ± 0.76	2.95 ± 0.76			
	intervention	2.93 ± 0.68	2.89 ± 0.61	2.85 ± 0.60			
	*t*	0.111	−0.280	−0.774	0.146	0.269	0.875
	*p*	0.912	0.780	0.440	0.704)	(0.765)	(0.419)
HDL-C	Control	1.27 ± 0.27	1.26 ± 0.32	1.26 ± 0.30			
	intervention	1.30 ± 0.30	1.33 ± 0.30	1.36 ± 0.30			
	*t*	0.537	1.129	1.837	1.737	2.076	3.530
	*p*	0.592	0.261	0.068	(0.192)	(0.134)	(0.035)

Remarks: interaction, intervention × time.

The results of the analysis of variance of repeated measurement data show the following:

1. intervention methods had statistically significant differences in the scores of self-management behavior and diet management, exercise management, emotional management, medication management and other lifestyle management (*p* < 0.05) (see Table 4).

Different intervention methods had statistical significance in body weight and WC (*p* < 0.05), but no statistical significance in blood pressure, FPG, and blood lipids (*p* > 0.05). The impact of community chronic disease management on the weight and WC of MS patients bereaved in the Wenchuan earthquake had an inter-group effect.

**Figure 1:**
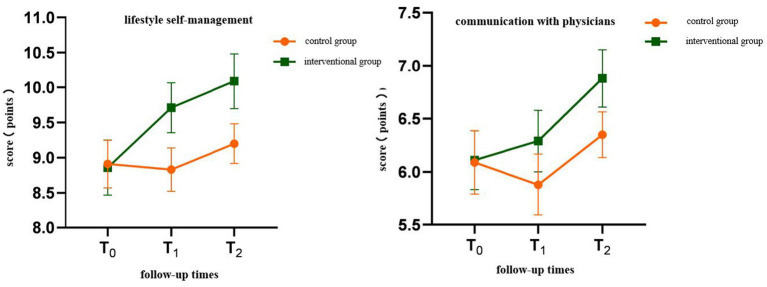
Interactive contour map of health self-management behavior score Interactive contour map of health self-management behavior score.

**Figure 2:**
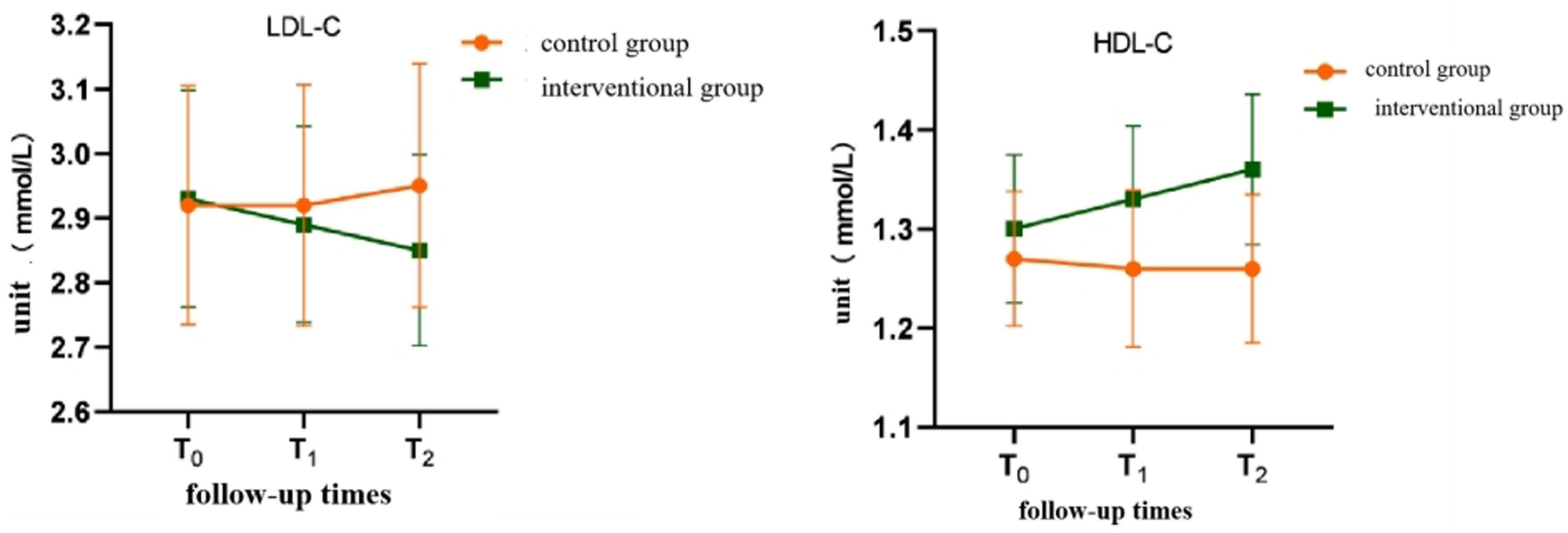
Interactive contour map of Metabolic Index.

1. Time effect: The differences in self-management behavior and scores of various dimensions at different follow-up time points were statistically significant (*p* < 0.05), showing a significant time effect (see Table 4). The differences in body weight, WC, BMI, and SBP at different follow-up time points were statistically significant (*p* < 0.05), showing a significant time effect (see Table 5).
2. Interaction effect between intervention and time: There was an interaction effect between different intervention methods and different follow-up times (*p* < 0.05). Through the interactive contour map (Figures 1 and 2), it can be seen that with the passage of follow-up time, the two groups of patients’ self-management behaviors and the trend of changes in the scores of each dimension are different (see Table 4, Figure 1).

Through the interactive contour map (see Figure 2), it can be seen that with the passage of follow-up time, the two groups of patients have different trends in weight, WC, BMI, blood pressure, FPG, and blood lipids.

1. Differences between groups: *t* tests were performed on the self-management behavior and scores of each dimension of the two groups of patients at each follow-up point. The results showed that at T0, the difference in scores between the two groups was not statistically significant (*p* > 0.05). The scores of the other dimensions at T1 and T2 were higher than those of the control group, and the differences were statistically significant (*p* < 0.05), see Table 4.

*T*-test was performed on the weight, WC, BMI, blood pressure, FPG and blood lipids of the two groups at each follow-up time point. The results showed: ① At T_0_, T_1_, and T_2_ three time points, there was a difference in BMI between the two groups. There was no statistical significance (*p* > 0.05); ②At T_0_, there was no significant difference in body weight and WC between the two groups (*p* > 0.05). ③There was no statistically significant difference in blood pressure and blood lipids between the two groups at the three time points (*p* > 0.05). ④At T_0_ and T_1_, there was no statistically significant difference in FPG between the two groups of patients (*p* > 0.05); at T_2_, the difference in FPG between the two groups was statistically significant (*p* < 0.05) (see Table 5).

## 4. Discussion

The results of this study showed that the total score of self-management behavior in the control group at T_2_ did not change much from the baseline, and the total score increased by an average of 1.18 points from the baseline, while the intervention group increased the self-management behavior at T_1_ and T_2_ (Figures 1, 2).

The results of this study are basically consistent with the relevant literature reports retrieved at home and abroad, and both indicate that patients can effectively improve their health self-management behaviors when they receive health self-management interventions. Such as Wang Yasha (20), Vanessa et al. (21), Marks et al. (22), Shin et al. (23) and Zhai Yingfen et al. (24).

The possible reasons for the expected results of this study are summarized into the following aspects. ① The study is designed on the basis of three theoretical frameworks combined with evidence-based guidelines, previous cross-sectional studies and qualitative interview results, and fully excavated MS self-management intervention methods are highly targeted. ② The design of the intervention plan of this study is based on the teaching of MS disease-related knowledge, starting with the patient’s cause, earthquake trauma/stress management, and sharing successful examples of communication with patients and empowering them. At the same time, it combines follow-up and strengthened interventions to health beliefs, produce a conscious and proactive attitude, and promote healthy behavior. ③ The researchers fully contacted and understanded the patients’ eating habits and cultural customs from current situation investigations and qualitative interviews. Solving the difficulties, needs and challenges encountered in the implementation of patients’ self-management behaviors is very important for improving patients’ health self-management behaviors, which is consistent with the research reports of Aktas and other scholars (25). ④ During the teaching intervention period, due to the impact of the epidemic, the hospital’s treatment process was accompanied by QR code scanning, body temperature measuring, nucleic acid testing, and patients and companions filling out the epidemiological questionnaire.

The results of this study showed that the weight of the subjects in the control group did not change much from the baseline at T_2_, and their weight increased by 0.68Kg on average from the baseline; while the subjects in the intervention group were significantly lower than the baseline at T_1_ and T_2_. The average weight loss was 4.26Kg, which was 6.0% less than the baseline weight. According to the evidence-based guidelines recommendation standard (19).

There are similarities and differences in the four results of blood lipids (TC, TG, HDL-C and LDL-C) reported by scholars Shengnan (26), Senhai et al. (27) and Yasha (20).

Analysis of the four blood lipids (TC, TG, HDL-C and LDL-C) between the two groups of patients in this study were not statistically significant at the two time points of T_1_ and T_2_. The possible reasons for the failure to achieve the expected results are: ① During the entire period of the intervention, the study was in the epidemic stage. It was forbidden to gather more 10 people, and collective entertainment activities (playing mahjong, dancing square dance) were canceled, which may be more idle than in previous years. ② The intervention of this study started in March. The climate became warmer and darker. Patients usually exercise moderately after eating. The work and rest habits of these two rural residents may be difficult to change within 5 months.

### 4.1. Struggles and limitations

Studies on the long-term impact of earthquake stress on the health of bereaved families are rarely reported worldwide. Due to some objective conditions in this study, the metabolic outcome indicators of this intervention study did not include HbA1c. The FPG results at the three time points of T_0_, T_1_, and T_2_ are used as indicators to judge whether the patient’s blood glucose has improved, rather than the average blood glucose HbA1c of 2 to 3 months, which may be biased.

This study selected three detection time points: baseline (T_0_), end of intervention (T_1_), and 2 months after the end of intervention (T_2_) on an evidence-based basis. The long-term effects of health self-management interventions need to be further explored. Small numbers examined and the consequent difficult generalisability of results.

## 5. Conclusion

The intervention program of healthy self-management for MS patients from bereaved families in the Wenchuan earthquake can effectively improve patients’ health self-management behaviors and patients’ weight, WC, BMI, and FPG in the short term. However, the effects of improving the patient’s blood lipid and blood pressure levels are uncertain and need further verification.

## Data availability statement

The original contributions presented in the study are included in the article/supplementary material, further inquiries can be directed to the corresponding author.

## Ethics statement

The studies involving humans were approved by Ethics Committee of West China Hospital of Sichuan University for approval(No:965). The studies were conducted in accordance with the local legislation and institutional requirements. Written informed consent for participation in this study was provided by the participants’ legal guardians/next of kin. Written informed consent was obtained from the minor(s)’ legal guardian/next of kin for the publication of any potentially identifiable images or data included in this article.

## Author contributions

ML: Conceptualization, Writing – original draft. JX: Software, Writing – review & editing. WS: Supervision, Writing – original draft. JN: Supervision, Writing – original draft.

## References

[1] Carmassi C Dell'Oste V Bertelloni CA. Disrupted rhythmicity and vegetative functions relate to PTSD and gender in earthquake survivors. Front Psychiatry. (2020) 16:492006. doi: 10.3389/fpsyt.2020.492006 PMC7701044 33304278

[2] Rafiey H Alipour F LeBeau R. Prevalence and determinants of PTSD 3 years after an earthquake in Iran. Community Ment Health J. (2019) 55:542–7. doi: 10.1007/s10597-019-00384-x 30796682

[3] State Council Information Office of the People's Republic of China, (2010), Wenchuan earthquake, Available at: http://www.scio.gov.cn/zhzc/6/2/Document/1003461/1003461.htm, (Accessed: October 14, 2009).

[4] Xinman D. A study on the bereavement, anxiety, depression and its influencing factors of the bereaved one year after the Wenchuan earthquake. China: Sichuan University (2010).

[5] Qianjin J. Medical psychology. Beijing: People's Health Publishing House (2000).

[6] Yongxiang M. Study on the effect of health self-management intervention for hypertension patients in Banfang community. China: Sichuan University (2010).

[7] Zhang G Cui P Jin W. Changes in hydrological behaviours triggered by earthquake disturbance in a mountainous watershed. Sci Total Environ. (2021) 18:1–6. doi: 10.1016/j.scitotenv.2020.143349 33168255

[8] Athyros VG Bouloukos VI Pehlivanidis AN Papageorgiou AA Dionysopoulou SG Symeonidis AN. The prevalence of the metabolic syndrome in Greece: the MetS-Greece multicentre study. Diabetes Obes Metab. (2005) 7:397–405. doi: 10.1111/j.1463-1326.2004.00409.x, PMID: 15955126

[9] Kohsari M Moradinazar M Rahimi Z. Liver enzymes and their association with some Cardiometabolic diseases: evidence from a large Kurdish cohort. Biomed Res Int. (2021) 2021:1–8. doi: 10.1155/2021/5584452 34235221 PMC8216792

[10] Nehus E. Obesity and chronic kidney disease. Curr Opin Pediatr. (2018) 30:241–6. doi: 10.1097/MOP.0000000000000586 29346138

[11] Xie K Bao L Jiang X. The association of metabolic syndrome components and chronic kidney disease in patients with hypertension. Lipids Health Dis. (2019) 18:229. doi: 10.1186/s12944-019-1121-5, PMID: 31881889 PMC6935087

[12] Gathirua-Mwangi WG Monahan PO Murage MJ Zhang J. Metabolic syndrome and total cancer mortality in the third national health and nutrition examination survey. Cancer Causes Control. (2017) 28:127–36. doi: 10.1007/s10552-016-0843-1, PMID: 28097473 PMC5308139

[13] Cooney LG Milman LW Hantsoo L Kornfield S Sammel MD Allison KC. Cognitive-behavioral therapy improves weight loss and quality of life in women with polycystic ovary syndrome: a pilot randomized clinical trial. Fertil Steril. (2018) 110:161–171.e1. doi: 10.1016/j.fertnstert.2018.03.028, PMID: 29908771 PMC6443091

[14] Shomaker LB Kelly NR Radin RM Cassidy OL Shank LM Brady SM. Prevention of insulin resistance in adolescents at risk for type 2 diabetes with depressive symptoms: 1-year follow-up of a randomized trial. Depress Anxiety. (2017) 34:866–76. doi: 10.1002/da.22617, PMID: 28370947 PMC5623599

[15] Williams J Stubbs B Gaughran F Craig T. 'Walk this Way' – a pilot of a health coaching intervention to reduce sedentary behaviour and increase low intensity exercise in people with serious mental illness: study protocol for a randomised controlled trial. Trials. (2016) 17:594. doi: 10.1186/s13063-016-1660-2, PMID: 27955680 PMC5154047

[16] Shan Q Shengli. A diagnostic test of the generalized anxiety scale for generalized anxiety disorder screening in the outpatient department of psychology in general hospitals. Chin J Ment Health. (2015) 29:939–44.

[17] Lee BJ Kim JY. Identification of metabolic syndrome using phenotypes consisting of triglyceride levels with anthropometric indices in Korean adults. BMC Endocr Disord. (2020) 20:29. doi: 10.1186/s12902-020-0510-0, PMID: 32103744 PMC7045372

[18] Zhihong N. A study on health self-management intervention in Xinjiang Kazakh patients with metabolic syndrome. China: Sichuan University (2019).

[19] Chinese Experts’. Consensus on Diagnosis and Treatment of Cardiovascular Disease with Insomnia (2017).

[20] Yasha W Xiao X Tao F. Study on the effect of comprehensive intervention in patients with metabolic syndrome. Chin J Dis Control. (2017) 21:784–8.

[21] Bullón-Vela V Abete I Tur JA Pintó X Corbella E Martínez-González MA. Influence of lifestyle factors and staple foods from the Mediterranean diet on non-alcoholic fatty liver disease among older individuals with metabolic syndrome features. Nutrition. (2020) 71:110620–8. doi: 10.1016/j.nut.2019.110620 31838461

[22] Marks S. Culturally sensitive education can decrease Hispanic workers’ risk of metabolic syndrome[J]. Workplace Health Saf. (2016) 64:543–9. doi: 10.1177/2165079916634712, PMID: 27059994

[23] Shin N-M Choi J Cho I Park BJ. Self-management program for heart healthy behavior among middle and old-aged Korean women at risk for metabolic syndrome. J Cardiovasc Nurs. (2017) 32:E8–E16. doi: 10.1097/JCN.0000000000000406 28306702

[24] Zhai Yingfen X Hongyan C. The effect of comprehensive nursing intervention on medication compliance, sleep quality and quality of life in patients with metabolic syndrome. China Med Herald. (2015) 12:142–5.

[25] Aktas MF Mahler A Hamn M Perger G Simon F Westenhöfer J. Lifestyle interventions in Muslim patients with metabolic syndrome-a feasibility study. Eur J Clin Nutr. (2018) 73:18–26. doi: 10.1038/s41430-018-0371-z 30538299

[26] Shengnan M. Community metabolic syndrome intervention research based on chronic disease management model. China: Zhejiang University (2017).

[27] Senhai Y Feixia P Hangjie G. Evaluation of the effect of intensive lifestyle intervention in rural metabolic syndrome patients. Prev Med. (2017) 29:1193–8.

